# Gastroprotective [6]-Gingerol Aspirinate as a Novel Aspirin-Derived Chemopreventive Agent Attenuating Colitis

**DOI:** 10.1021/acs.jafc.5c17778

**Published:** 2026-04-20

**Authors:** Pei-Sheng Lee, Shuwei Zhang, Yingdong Zhu, Vadin Ha, Shengmin Sang

**Affiliations:** † Laboratory for Functional Foods and Human Health, Center for Excellence in Post-Harvest Technologies, 3616 North Carolina Agricultural and Technical State University, Kannapolis, North Carolina 28081, United States; ‡ Center for Gastrointestinal Biology and Disease, University of North Carolina at Chapel Hill, Chapel Hill, North Carolina 27599, United States

**Keywords:** [6]-gingerol aspirinate, aspirin, colitis, anti-inflammatory activity, metabolomics, biomarkers

## Abstract

Daily low-dose aspirin reduces cardiovascular disease and colorectal cancer risk, but it is limited by gastrointestinal toxicity. To enhance safety and efficacy, we developed [6]-gingerol aspirinate (GAS) by conjugating aspirin with ginger-derived bioactive [6]-gingerol. This study evaluated GAS in a dextran sodium sulfate (DSS)-induced colitis mouse model. GAS significantly improved clinical symptoms and reduced pro-inflammatory markers (IL-6, TNF-α, IL-1β, TGF-β, and COX-2), whereas aspirin alone showed minimal protection. Untargeted LC/MS-based metabolomics revealed that GAS reversed 73 DSS-altered metabolites, including those related to lipids, amino acids, and carbohydrate metabolisms, many previously linked to inflammatory bowel disease and colorectal cancer. Correlation analysis showed strong associations between these metabolites and inflammatory cytokines, suggesting their involvement in GAS-mediated anti-inflammatory effects. Overall, GAS provides superior protection against colitis through coordinated suppression of inflammation and modulation of disease-associated metabolic pathways, highlighting its potential as a safer chemopreventive agent and identification of candidate metabolic biomarkers.

## Graphical abstract

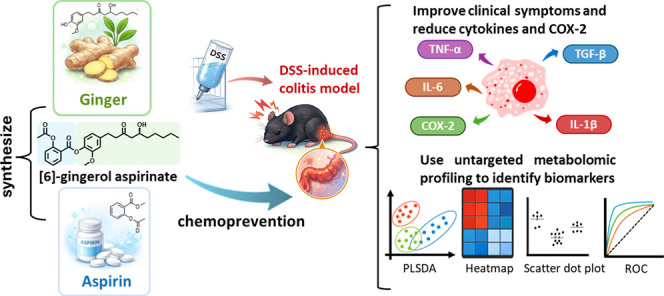

## Introduction

Aspirin, a nonsteroidal anti-inflammatory drug (NSAID), has a broad spectrum of applications and effects across various disease categories. Low-dose aspirin (75–325 mg/day) has been demonstrated to prevent cardiovascular disease in high-risk patients.^1^ However, it was not until recent decades that the potential of aspirin to reduce the occurrence and mortality of cancer, particularly colorectal cancer (CRC),^2^ became apparent. However, a significant drawback of aspirin usage is its propensity to induce ulceration and bleeding in the gastrointestinal tract.^3^ Aspirin, by blocking Cyclooxygenase-1 (COX-1), exerts an antiplatelet effect but also inhibits the gastroprotective effects of Prostaglandin E_2_ (PGE_2_) and PGI_2_, thereby increasing the risk of gastrointestinal complications, such as gastric ulcers and bleeding.

Ginger is the rhizome of a perennial herb belonging to the Zingiberaceae family and possesses a distinctive pungent taste and warming properties. Traditionally, ginger, with [6]-gingerol being the most abundant and bioactive compound, has been employed to alleviate symptoms such as colds, vomiting, phlegm, and cough. Recent research has unveiled a broad spectrum of pharmacological effects associated with ginger, including anti-inflammatory, cardiotonic, antioxidant, antitumor, and immune-enhancing properties. Consequently, ginger has emerged as a potential treatment option for various conditions such as rheumatic pain, arthritis, sore throat, and chronic vomiting, among others.^4^

Preclinical studies have demonstrated that compounds found in ginger can alleviate gastric ulceration. Our laboratory has developed and synthesized a novel dietary derivative of aspirin, known as [6]-gingerol aspirinate (GAS).^3^ Our previous data indicate that GAS exhibits enhanced anticancer properties *in vitro* and demonstrates superior gastroprotective effects in mice. GAS has also shown resilience to stomach acid, and it decomposes in the intestinal linings, releasing both aspirin and [6]-gingerol simultaneously. Additionally, we found that GAS effectively inhibits both COX-1 and COX-2. Our findings highlight the enhanced anticancer properties and gastroprotective effects of GAS, suggesting its potential as a therapeutic alternative to aspirin in inflammatory diseases without adverse effects on stomach mucosa.^3^

Ulcerative colitis (UC) is a chronic inflammatory bowel disease (IBD) that primarily affects the colon and rectum, with its exact cause remaining unknown. The pathogenesis of UC is believed to involve various factors, including genetic predisposition, environmental influences, luminal factors, and dysregulation of mucosal immunity. UC has emerged as a significant global health concern due to its high prevalence in developed nations and a notable rise in incidence rates observed in developing regions.^5^

Dextran sodium sulfate (DSS)-induced colitis is one of the most widely used and well-established animal models for studying intestinal inflammation. When administered in drinking water, DSS disrupts the epithelial barrier of the colon, allowing luminal antigens and microbial components to penetrate underlying tissues and trigger a robust inflammatory response.^6^ This model closely resembles several pathological features of human ulcerative colitis, including body weight loss, diarrhea, rectal bleeding, mucosal ulceration, and marked increases in pro-inflammatory cytokines.^7,8^ Because of its reproducibility, simplicity, and strong inflammatory phenotypes, the DSS model is commonly used to evaluate the efficacy of anti-inflammatory agents and to investigate molecular mechanisms underlying intestinal injury and repair.^9^

The aim of this study was to determine the protective effects of the novel aspirin-dietary derivative GAS on DSS-induced colitis in mice and to compare its efficacy with that of aspirin. In addition, we investigated the underlying metabolic mechanisms of GAS-mediated protection using an untargeted metabolomic approach and assessed correlations between GAS-responsive metabolites and inflammatory cytokines.

## Materials and Methods

### Chemicals and Reagents

DSS (mol wt, 36–50 kDa) was purchased from MP Biomedicals. GAS was synthesized in our laboratory.^3^ The mouse anti-β-actin monoclonal antibody (catalog number: 4970S) was purchased from Cell Signaling Technology (Danvers, MA, USA). The anti-COX-2 monoclonal antibody (catalog number: 160112) was purchased from Cayman Chemical (Ann Arbor, MI, USA).

### Animal Experimental Design

Male C57BL/6J mice, aged 6 weeks, were procured from the Jackson Laboratory. They were housed under controlled conditions of temperature (20 ± 2 °C) and relative humidity (50 ± 10%), with a 12 h light–dark cycle. All animal experimental procedures were approved by the Institutional Animal Care and Use Committee of the North Carolina Research Campus (Protocol #20-007). To account for potential mortality during DSS induction, the DSS-treated group included 12 mice, whereas all other experimental groups consisted of 8 mice each, as specified in the figure legends. The mice were randomly divided into five groups: ND (normal diet), DSS (ND + 2.5% DSS), DSS + LGAS (0.5 mmol GAS + 2.5% DSS), DSS + HGAS (1.5 mmol GAS + 2.5% DSS), and DSS + AS (1.5 mmol aspirin + 2.5% DSS). AIN-93G served as the base experimental diet. The selection of these two doses was based on clinically recommended aspirin dosing. Human equivalent doses for the two doses used in this study were calculated using body surface area normalization in accordance with FDA guidance, applying a mouse-to-human conversion factor of 12.3 (*K*_m_ factor: mouse = 3, human = 37). After applying this conversion, the low dose of 0.5 mmol of GAS administered to mice corresponds to 0.5 mmol of aspirin equivalents, equivalent to 52.8 mg of aspirin. The high dose of 1.5 mmol of GAS delivers 1.5 mmol of aspirin equivalents, corresponding to 158.4 mg of aspirin. Accordingly, both the low and high GAS doses yield aspirin-equivalent exposures that fall within or below the clinically relevant low-dose aspirin range of 75–325 mg/day for adults.^1^ The colitis model was induced by treatment with 2.5% DSS for 5 days, followed by a period of 7 days of water for recovery. Mice in the treated groups received pretreatment with GAS or aspirin for 2 weeks. The experimental design is summarized in Figure 1A. The animals had ad libitum access to food and water. Mice scheduled for sacrifice were anesthetized, blood was collected by cardiac puncture, and organs were immediately harvested, weighed, and frozen. All samples were stored at −80 °C until analysis.

**Figure 1:**
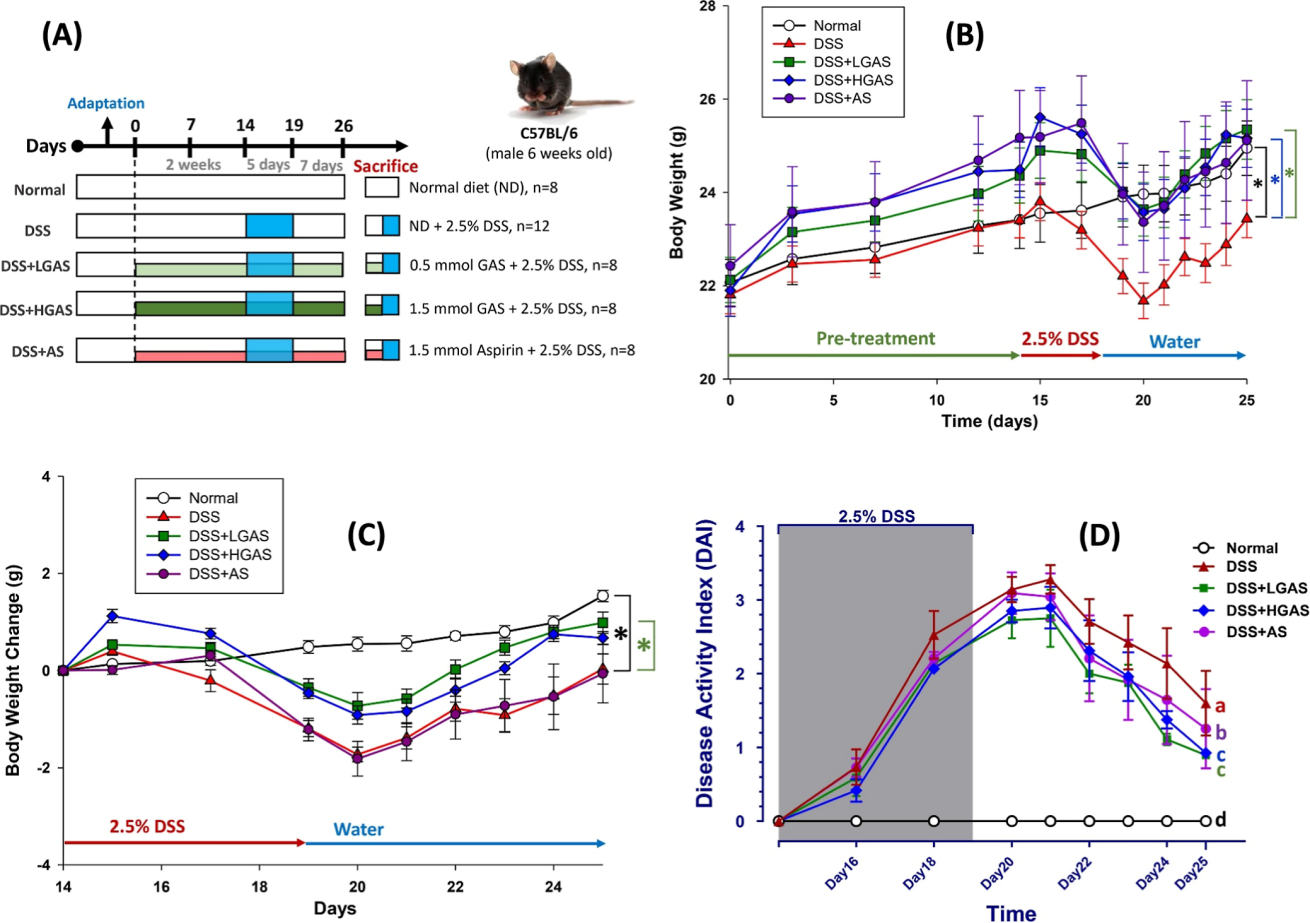
Experimental protocol and general animal status. (A) Schema of DSS-induced colitis mice model and experiment design. (B) Body weight over the period of experiment. (C) Body weight change after DSS treatment. (D) Disease activity index (DAI) over the period of experiment. For panels B and C, comparisons between two groups were analyzed using Student’s *t*-test. For panel D statistical significance was determined by one-way ANOVA followed by Tukey’s post hoc test. The values with different letters are significantly different (*p* < 0.05) between each group. Data are presented as mean ± SE (*n* = 8 or 12 per group).

### Disease Activity Index and Cytokine Analysis

To assess the severity of colitis, the disease activity index (DAI) was calculated based on three parameters: (i) body weight loss, (ii) fecal blood, and (iii) stool consistency. Body weight loss was scored as follows: 0: none; (1:1–5%; 2:5–10%; 3:10–15%; and 4: >15%). Fecal blood was scored as 0, negative; 1, Hemoccult positive; 2, Hemoccult positive with visible blood; 3, moderate bleeding; and 4, gross bleeding. Stool consistency was scored as 0, normal; 1, moist or sticky stool; 2, soft stool; 3, soft stool with mild diarrhea; and 4, diarrhea. Body weight loss was calculated as the percentage change relative to the initial body weight on day 0. Occult blood was detected using the Hemoccult Guaiac Fecal Occult Blood Test kit (Beckman Coulter) following the manufacturer’s instructions.

Cytokine levels were measured using ELISA kits specific for each cytokine: IL-6 (OptEIA Mouse IL-6 ELISA Kit, from Becton Dickinson), TGF-β (Mouse TGF beta 1 ELISA Kit, from Invitrogen), IL-1β (Mouse IL-1 beta Quantikine ELISA Kit, from R&D system), and TNF-α (Mouse TNF-alpha Quantikine ELISA Kit, from R&D system), according to the manufacturer’s protocols.

### Western Blot Analysis

For protein analysis, the entire colon was homogenized on ice using a bead mill homogenizer (OMNI International) and lysed with ice-cold lysis buffer (Cell Signaling Technology). The resulting mixture was then centrifuged at 16,500 g for 30 min at 4 °C. The protein content was quantified using a Pierce BCA Assay Kit (Thermo Fisher Scientific). Twenty-five micrograms of total protein was loaded onto SDS-polyacrylamide gels and transferred to polyvinylidene fluoride (PVDF) membranes. After blocking with milk (Bio-Rad Laboratories) for 1 h at room temperature, the membranes were incubated overnight with COX-2 primary antibodies. Subsequently, the blots were washed with TBS-Tween 20 and probed with the appropriate secondary antibody horseradish peroxidase (1:5000) for 1 h. Protein bands were visualized using chemiluminescence with a West Femto maximum detection substrate (Thermo Fisher Scientific). To ensure equal protein loading in each lane, β-actin was used as the loading control. The fold-induction of proteins was calculated by normalizing the intensity of the band of interest to β-actin first and then to control lanes using ImageJ imaging software (National Institutes of Health).

### Preparation of Metabolomic Samples

One hundred microliters of plasma was mixed with 500 μL of ice-cold methanol containing cholesterol-d6, tridecanoic acid, DL-4-chlorophenylalanine, and DL-2-(4-chlorophenyl)­glycine to precipitate proteins, followed by vortexing for 1 min. The mixture was then sonicated for 5 min and centrifuged at 15,000 g for 15 min at 4 °C to collect the supernatant. The supernatant was dried using a SpeedVac and reconstituted in 100 μL of 50% methanol containing 21 isotopically labeled internal standards, each at a concentration of 500 nM, as described previously.^10^ A method blank was prepared using the same solvents, consumables, and procedures without plasma. A quality control (QC) sample was prepared by pooling 20 μL of each sample. QC samples were analyzed to monitor drift, distinguish between low- and high-quality data, and stabilize the analytical platform.

### Metabolomic Data Acquisition

Metabolomic data were collected using our established LC–MS/MS workflow with consistent chromatographic conditions and data-dependent acquisition parameters, enabling direct annotation with our in-house spectral library.^10^ Briefly, mass spectra were collected on a Q Exactive Plus Orbitrap system coupled to a Vanquish UPLC instrument (Thermo Scientific). For normal-polarity metabolites, positive-mode data were acquired after separation using a Waters BEH C18 column with an acetonitrile/water containing 0.1% formic acid mobile-phase system, while negative-mode data were acquired after separation using another Waters BEH C18 column with an ammonium bicarbonate/methanol/water system. For high-polarity metabolites, negative-mode data were collected after separation using a Waters BEH amide column with an ammonium formate/acetonitrile/methanol/water system.

### Metabolomic Data Processing, Multivariate Analysis, and Pathway Analysis

The raw data acquired with three LC–MS conditions were processed separately with MS DIAL (ver. 4.9) for data collection, peak detection and alignment, and compound identification. Compound identification was performed using our newly updated in-house library and online databases. After processing, the alignment results were explored to sort out the peak heights of the annotated metabolites for statistical analysis. The missing values were replaced with 1/10 of the minimum peak height over all samples.

Before multivariate analysis, the peak heights of all annotated metabolites in all samples and QCs were normalized by SERRF (created by Shiny R. at UC Davis). Multivariate analysis was then performed using the web-based metabolomics platform MetaboAnalyst 6.0. Data were log_10_-transformed and autoscaled. Supervised exploratory analysis was conducted using orthogonal partial least-squares-discriminant analysis (OPLS-DA). ANOVA was used to determine variance, with FDR-adjusted *p*-values lower than 0.05 considered indicative of significant biomarkers. The biological pathway analysis was performed on MetaboAnalyst 6.0 using HMDB IDs for all identified potential biomarkers.

### Statistical Analysis

Statistical analyses were performed using Student’s *t*-test for comparisons between two groups or one-way ANOVA followed by Tukey’s post hoc test for comparisons involving more than two groups. One-way ANOVA conducted using SPSS (version 21). Outlier detection was performed prior to statistical analysis. Outliers were identified using an interquartile range (IQR)-based approach in Microsoft Excel, following established procedures for outlier detection. Identified outliers were excluded from subsequent analyses to ensure the robustness of the statistical results. Data were expressed as the mean ± standard error (SE) for the specified number of independent experiments, and *p*-values <0.05 were considered statistically significant.

Correlations between colon pro-inflammatory markers and plasma metabolites from untargeted metabolomic profiling were assessed using Spearman’s rank correlation with two-tailed testing in GraphPad Prism (version 10, GraphPad Software, San Diego, CA, USA). Data from all groups (Normal, DSS, DSS + LGAS, DSS + HGAS, and DSS + aspirin) were analyzed together. A correlation matrix was generated to visualize the relationships between variables, and statistical significance was set at *p* < 0.05. A heatmap was generated to present the results based on the correlation coefficient (r) values.

Multivariate receiver operating characteristic (ROC) analysis was performed to evaluate the discriminatory performance of metabolomic profiles between experimental groups using pairwise comparisons (ND vs DSS, HGAS vs DSS, and HGAS vs AS). ROC modeling was conducted with Monte Carlo cross-validation (MCCV) using balanced subsampling. In each iteration, two-thirds of the samples were randomly selected to build the classification model, and the remaining one-third were used for validation. This procedure was repeated multiple times to estimate the model stability and predictive performance. Feature ranking was performed based on variable importance, and classification models were constructed using increasing numbers of top-ranked metabolites (3, 5, 10, 20, 36, and 73 variables). Model performance was evaluated by calculating the area under the ROC curve (AUC) along with the corresponding 95% confidence intervals (CI). Feature selection frequency was additionally calculated to assess the stability of metabolite inclusion across cross-validation iterations. Metabolites with a high selection frequency were considered stable contributors to group discrimination.

## Results

### GAS Markedly Ameliorates DSS-Induced Colitis and Outperforms Aspirin in Mice

As shown in Figure 1B,C, DSS administration markedly reduced body weight, with the DSS group exhibiting the greatest weight loss, approximately 9.5% lower than the Normal group on day 20. Mice treated with low- or high-dose GAS maintained significantly higher body weights than the DSS group, showing 8.2% and 7.4% greater body weight, respectively. In contrast, aspirin treatment did not prevent DSS-induced body weight loss. The maximum body weight reduction reached 1.7 g in the DSS group and 1.8 g in the aspirin group, whereas mice treated with low- and high-dose GAS lost only 0.7 and 0.9 g, respectively.

Disease activity index (DAI) scores are presented in Figure 1D. The DSS group showed the highest DAI scores. Both low- and high-dose GAS significantly reduced DAI scores by 44% and 42%, respectively, compared to DSS. Aspirin treatment resulted in a 21.6% reduction. DAI scores in the aspirin group remained significantly higher than those in both GAS-treated groups.

Colon length measurements (Figure 2A,B) showed significant shortening in DSS-treated mice (5.2 ± 0.11 cm). Treatment with LGAS (5.9 ± 0.11 cm) and HGAS (5.9 ± 0.22 cm) significantly restored the colon length. Aspirin treatment also increased colon length (5.7 ± 0.14 cm) compared with DSS.

**Figure 2:**
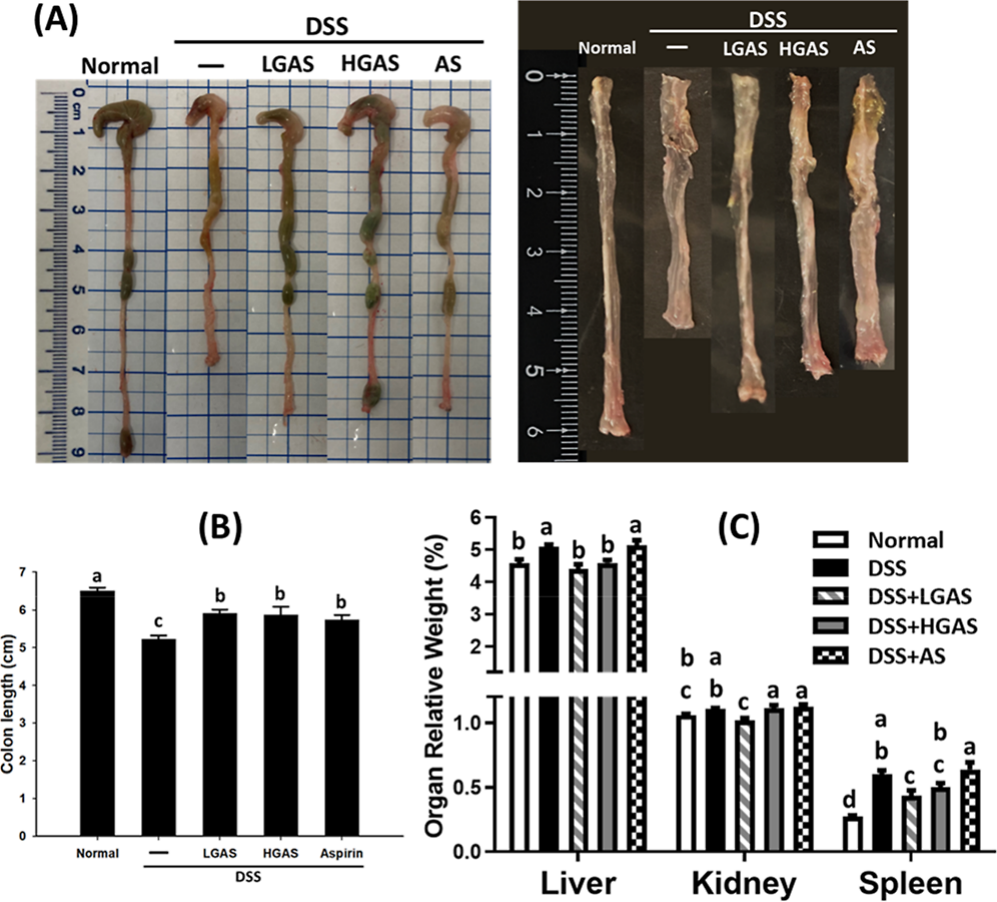
Effect of GAS on colon length and organ relative weight in DSS-induced C57BL/6 mice. (A) Representative macroscopic view of the colon of different experimental diets after DSS treatment. (B) Colon length. (C) Relative organ weight. Data are expressed as means ± SE (*n* = 7–12 per group, one sample was excluded after outlier analysis). The significance of differences among the five groups was analyzed by one-way ANOVA and Tukey’s multiple range tests. The values with different letters are significantly different (*p* < 0.05) between each group.

Relative organ weights are listed in Figure 2C. DSS treatment increased relative liver (∼10%) and spleen (∼55%) weights compared with the Normal group (5.1 ± 0.08% and 0.6 ± 0.03%, respectively). Low-dose GAS reduced liver and spleen weights to 4.4 ± 0.14% and 0.4 ± 0.04%, respectively. Aspirin-treated mice showed no significant improvement, with liver and spleen weights remaining elevated (5.1 ± 0.16% and 0.6 ± 0.06%).

Supporting Information, Figure S1, shows food and liquid intake during the experiment. Except for transient fluctuations during the DSS induction period, no significant differences were observed among the groups.

### GAS Modulates Inflammation-Related Cytokine Responses in DSS-Induced Colitis

Cytokine levels in plasma and colon tissue are listed in Figure 3. In plasma, DSS treatment significantly increased IL-6 and TNF-α levels compared with the Normal group. High-dose GAS significantly reduced plasma IL-6 and TNF-α levels by 29.4% and 14.7%, respectively, relative to DSS. Aspirin treatment did not significantly alter these cytokines. Plasma IL-1β levels were low across all groups. Plasma TGF-β levels were reduced in the DSS group compared with the Normal group.

**Figure 3:**
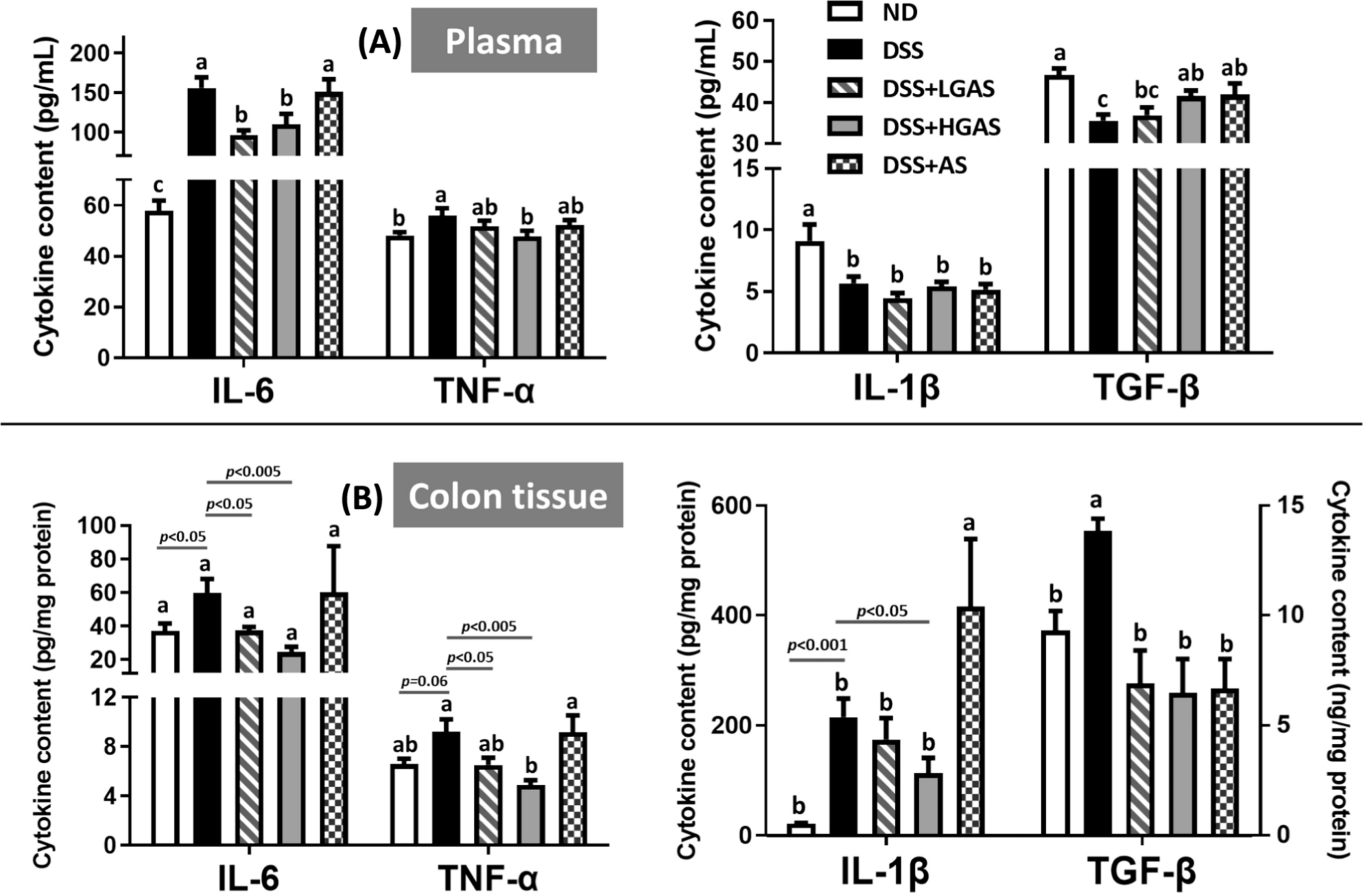
Effect of GAS on the production of inflammation-related cytokines. Cytokine levels in (A) plasma and (B) colon tissues. Statistical significance was determined by one-way ANOVA followed by Tukey’s post hoc test. The values with different letters are significantly different (*p* < 0.05) between each group. Comparisons between two groups were analyzed using Student’s *t*-test. Data are presented as mean ± SE (*n* = 6–12 per group, two samples were excluded after outlier analysis).

In the colon tissue, DSS treatment significantly elevated IL-6 and TNF-α levels. High-dose GAS markedly reduced colonic IL-6 and TNF-α levels by 59.1% and 46.9%, respectively. Aspirin treatment did not produce a significant improvement and showed substantial intragroup variability. Colonic IL-1β levels were markedly increased following DSS induction, with approximately a 10-fold elevation compared with that of the Normal group. High-dose GAS reduced IL-1β levels by 47.3%, whereas aspirin treatment resulted in levels nearly 1.9-fold higher than those observed in the DSS group. DSS treatment significantly increased the colonic TGF-β levels. Both GAS and aspirin treatments reduced colonic TGF-β levels compared with DSS.

### GAS Reduces DSS-Induced COX-2 Expression in the Colon

COX-2 in colon tissue is shown in Figure 4. DSS treatment significantly increased COX-2 expression by 2.1-fold compared with the Normal group. Aspirin treatment reduced COX-2 expression by approximately 70% relative to DSS; however, this reduction did not reach statistical significance. In contrast, both low- and high-dose GAS significantly reversed the DSS-induced increase, reducing COX-2 expression to levels below those observed in the Normal group.

**Figure 4:**
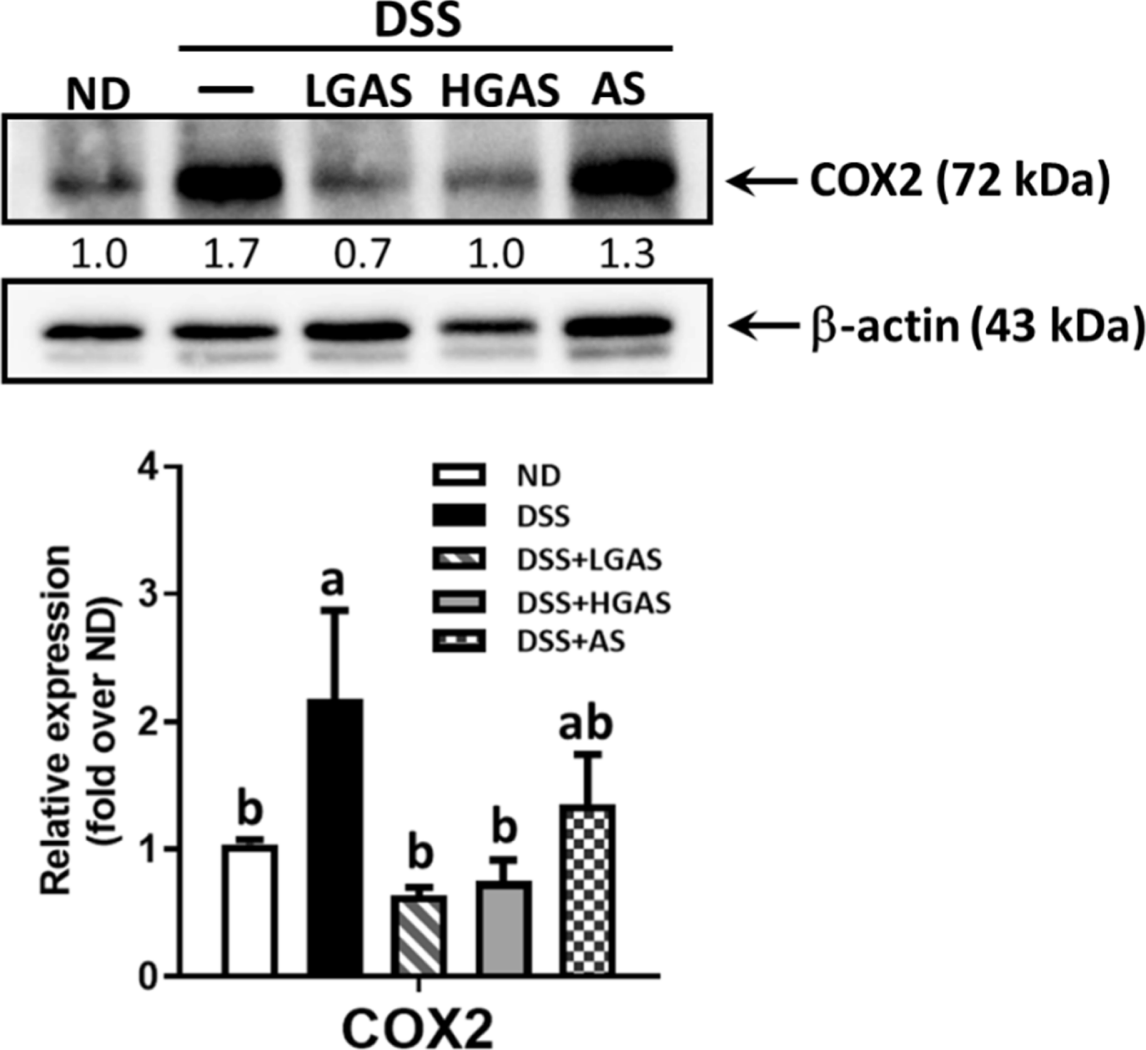
GAS downregulates the COX-2 levels in DSS-induced colitis. The protein levels of COX-2 were analyzed by Western blot using β-actin as the loading control. Quantification of the relative protein level was normalized with β-actin, and the results are shown as a bar graph. Data are expressed as means ± SE (*n* = 8 or 12 per group). The significance of differences among the five groups was analyzed by one-way ANOVA and Tukey’s multiple range tests.

### Using Untargeted Metabolomic Profiling to Identify Biomarkers Associated with the Protective Effects of GAS in DSS-Induced Colitis

Untargeted LC/MS-based metabolomic analysis identified 1794 metabolites across all plasma samples (without deduplication). Data were processed using MetaboAnalyst v. 6.0. PLS-DA demonstrated clear separation between the ND and DSS groups, indicating marked metabolic alterations induced by DSS (Figure 5A). The GAS-treated group exhibited a metabolic profile shifted toward that of the ND group, whereas the aspirin group clustered more closely with the DSS group.

**Figure 5:**
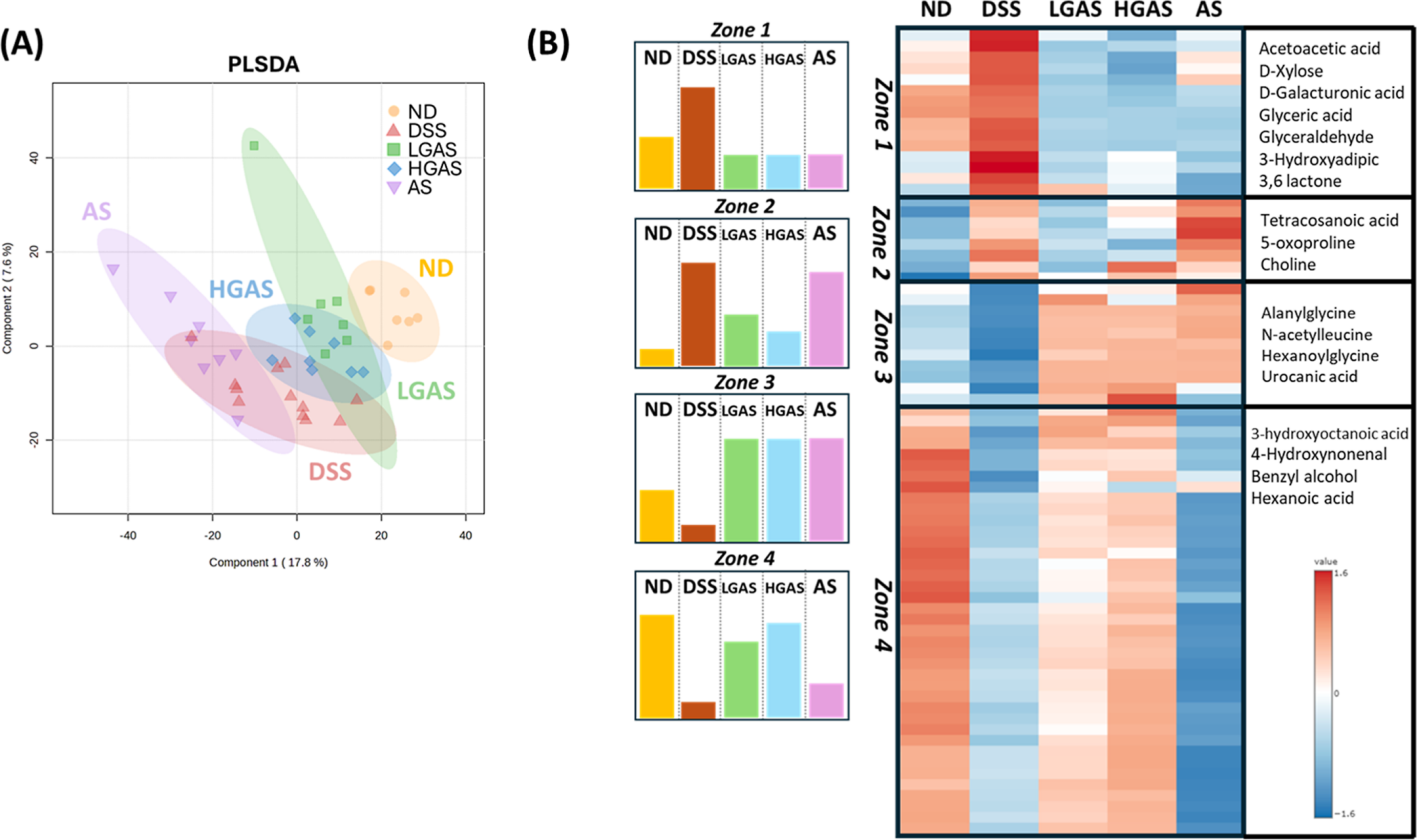
Metabolomic analysis. (A) PLSDA score plot. Yellow dots stand for the Normal group, red dots stand for the DSS group, green dots stand for the low-dose GAS group, blue dots stand for the high-dose GAS group, and purple dots stand for the aspirin group. PC 1 (*x*-axis) and PC 2 (*y*-axis) accounted for 37.2% of the total variance. (B) Hierarchical clustering heatmap for the 73 metabolites using *p*-value criteria. Each row indicates a metabolite, and each column indicates the group average. The correlation degrees are shown using a color scale; the red color represents upregulation, while the blue color represents downregulation (*n* = 8 or 12 per group).

One-way ANOVA with Tukey’s post hoc test (*p* < 0.05) identified 1152 metabolites that differed significantly among groups. Among these, 217 metabolites were significantly altered between the ND and DSS groups. Of the 217 metabolites, 73 were significantly reversed by GAS treatment. Heatmap analysis categorized the 73 GAS-responsive metabolites into four zones based on response patterns (Figure 5B): zone 1, metabolites elevated by DSS and reversed by both GAS and aspirin; zone 2, metabolites elevated by DSS and selectively reversed by GAS; zone 3, metabolites reduced by DSS and restored by both GAS and aspirin; zone 4, metabolites reduced by DSS and selectively restored by GAS.

Among the 73 GAS-responsive metabolites, 45 were successfully matched in pathway analysis (including four manually verified matches) and are summarized in Table 1. Lipid-derived metabolites constituted the largest category (*n* = 14). Of the 45 metabolites, 23 have been previously reported in association with colitis or colorectal cancer. Seventeen representative metabolites (11 identified using the in-house library and 6 with excellent public MS/MS matches) are shown in scatter plots in Figure 6.

**Table 1:** List of Selected Significant Metabolites from All Identified Metabolites Analyzed by Tukey’s Post Hoc Analysis

class	subclass	metabolite	HMDB	PubChem	*p*-value
lipid	short-chain fatty acids	propionic acid	HMDB0000237	1032	2.13 × 10^–5^
lipid	medium-chain fatty acids	(+)-abscisic acid	HMDB0035140	5702609	3.67 × 10^–15^
lipid	medium-chain fatty acids	(2E,4E)-hexa-2,4-dienoic acid	HMDB0029581	643460	2.25 × 10^–6^
lipid	medium-chain fatty acids	2-octenoic acid	HMDB0000392	5282713	3.93 × 10^–7^
lipid	medium-chain fatty acids	3-hydroxyoctanoic acid	HMDB0001954	11367166	2.38 × 10^–7^
lipid	medium-chain fatty acids	6-heptynoic acid		4377950	9.71 × 10^–6^
lipid	medium-chain fatty acids	dodec-2-enedioic acid	HMDB0340663		2.11 × 10^–7^
lipid	medium-chain fatty acids	hexanoic acid	HMDB0000535	8892	1.14 × 10^–6^
lipid	medium-chain fatty acids	sebacic acid monomethyl ester	HMDB0341393		3.86 × 10^–5^
lipid	medium-chain fatty acids	undecylenic acid	HMDB0033724	5634	7.71 × 10^–9^
lipid	methyl-branched fatty acids	2-propyl-4-pentenoic acid	HMDB0013897	104896	1.43 × 10^–4^
lipid	long-chain fatty acids	tetracosanoic acid	HMDB0002003	11197	8.97 × 10^–16^
lipid	fatty alcohols	4-hydroxynonenal	HMDB0004362	5283344	1.54 × 10^–5^
lipid		prostaglandin D_2_ methyl ester			5.05 × 10^–5^
amino acid	l-glutamic acid	5-oxoproline	HMDB0000267	7405	5.65 × 10^–7^
amino acid	dipeptides	alanylglycine	HMDB0006899	6998028	2.09 × 10^–23^
amino acid	dipeptides	Ile–Ile	HMDB0028910	13879965	7.48 × 10^–6^
amino acid	L-alpha-amino acids	methionine sulfone	HMDB0062174	445282	3.93 × 10^–3^
amino acid	*N*-acyl-alpha amino acids	hexanoylglycine	HMDB0000701	99463	4.89 × 10^–7^
amino acid	*N*-acyl-alpha amino acids	*N*-acetylleucine	HMDB0011756	70912	3.30 × 10^–7^
amino acid	serine and derivatives	serine	HMDB0000187	5951	3.07 × 10^–14^
amino acid	organosulfonic acids	taurine	HMDB0000251	1123	1.29 × 10^–13^
carbohydrate	monosaccharides	glyceraldehyde	HMDB0001051	751	1.85 × 10^–5^
carbohydrate	disaccharides	d-turanose	HMDB0011740	5460935	1.29 × 10^–16^
carbohydrate	pentoses	d-xylose	HMDB0000098	135191	7.27 × 10^–24^
organic acid	short-chain keto acids	2-oxobutyric acid	HMDB0000005	58	4.88 × 10^–7^
organic acid	short-chain keto acids	acetoacetic acid	HMDB0000060	96	7.17 × 10^–25^
organic acid	hippuric acids	2-hydroxybenzoylaminoacetic acid	HMDB0000840	10253	5.88 × 10^–3^
organic acid	hippuric acids	3-methoxy-4-hydroxyhippuric acid	HMDB0060026	3083688	1.30 × 10^–5^
organic acid	sugar acids	d-galacturonic acid	HMDB0002545	84740	2.45 × 10^–21^
organic acid	sugar acids	glyceric acid	HMDB0000139	439194	8.01 × 10^–6^
organic acid	jasmonic acids	jasmonic acid	HMDB0032797	5281166	1.50 × 10^–6^
organic acid	hydroxybenzoic acid derivatives	3-hydroxyanthranilic acid	HMDB0001476	86	9.87 × 10^–8^
organic acid	tricarboxylic acids and derivatives	cis-aconitic acid	HMDB0000072	643757	1.01 × 10^–21^
organic acid	imidazolyl carboxylic acids and derivatives	urocanic acid	HMDB0000301	736715	8.13 × 10^–6^
21-hydroxysteroids		cortisol	HMDB0000063	5754	4.07 × 10^–5^
amine		1-methylhistamine	HMDB0000898	3614	4.85 × 10^–5^
alcohols and polyols		1,4-butynediol	HMDB0245045	8066	1.06 × 10^–26^
aromatic alcohol		benzyl alcohol	HMDB0003119	244	1.03 × 10^–5^
benzenoids		[6]-paradol	HMDB0030801	94378	2.01 × 10^–19^
bilirubins		stercobilin	HMDB0240259	44457542	8.61 × 10^–4^
cholines		choline	HMDB0000097	305	3.51 × 10^–8^
gamma butyrolactones		dehydroascorbic acid	HMDB0001264	440667	1.40 × 10^–20^
hydroquinolones		4-hydroxyquinoline	HMDB0246466		1.10 × 10^–8^
		3-hydroxyadipic acid 3,6-lactone			2.28 × 10^–6^

**Figure 6:**
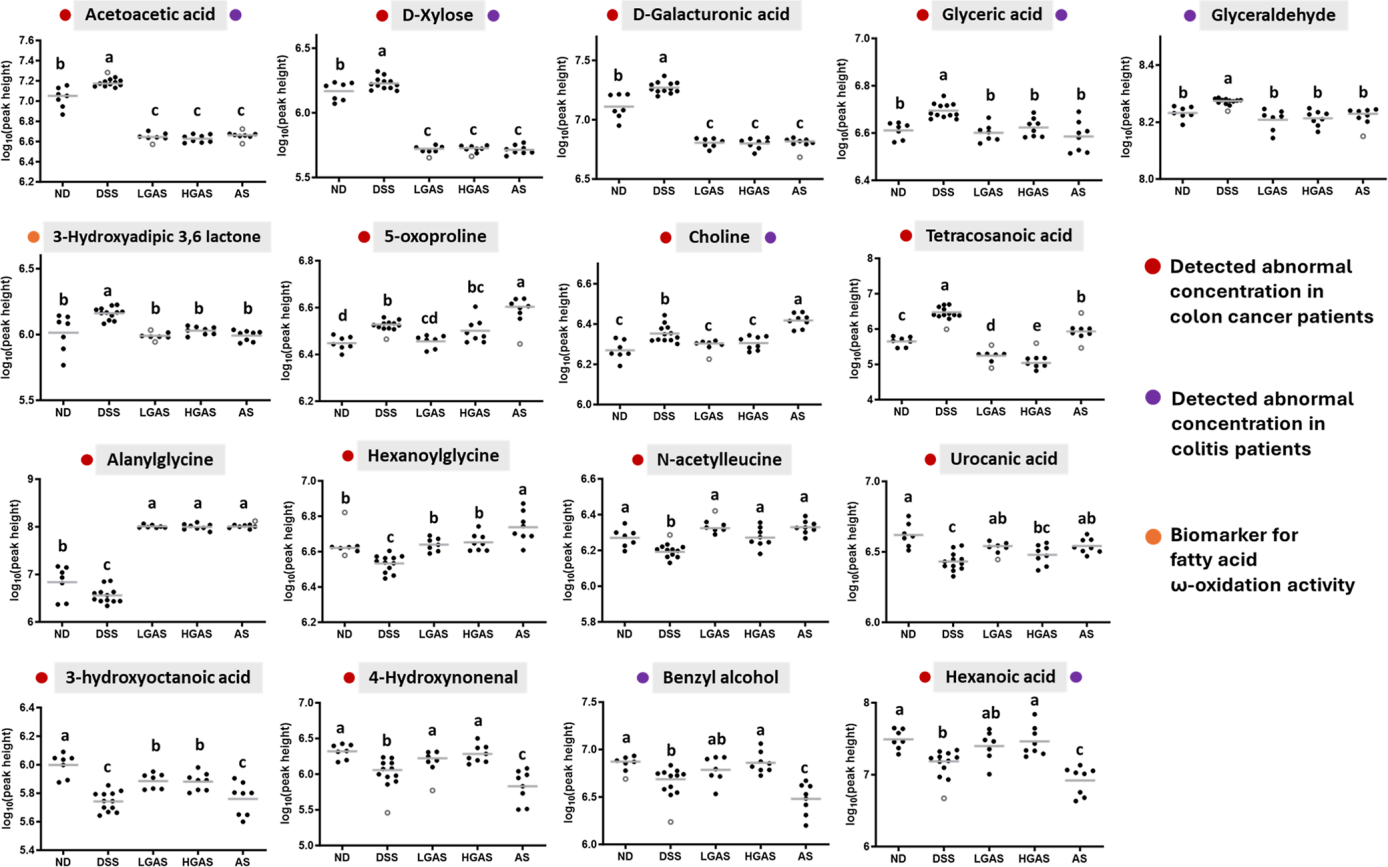
Scatter dot plot of important metabolites related to intestinal function. The significance of differences among the five groups was analyzed by one-way ANOVA and Tukey’s multiple range tests. The values with different letters are significantly different (*p* < 0.05) between each group. Hollow dots stand for outliers (*n* = 6–12 per group; samples were excluded following outlier analysis according to the predefined statistical criteria).

### Correlation Analysis between Colon Pro-Inflammatory Markers and Metabolites Identified from Untargeted Metabolomics

Correlation analysis was performed between 17 representative metabolites and colonic cytokines (IL-6, TNF-α, IL-1β, and TGF-β). As shown in Figure 7, 16 of the 17 metabolites, except urocanic acid, were significantly correlated to at least one cytokine. Several metabolites exhibited strong positive correlations with pro-inflammatory cytokines, whereas others showed inverse correlations. These associations indicate a close relationship between the identified metabolites and colonic inflammatory status.

**Figure 7:**
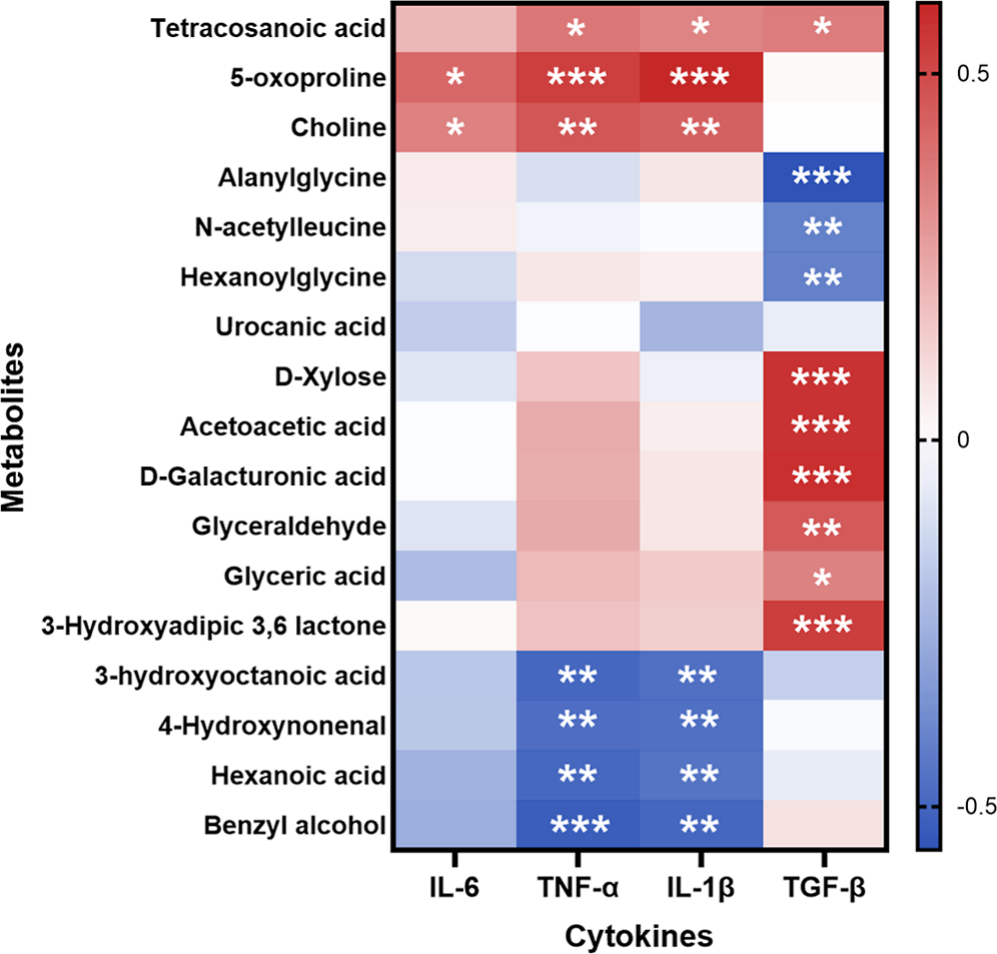
Correlation analysis between metabolites in plasma and inflammation-related cytokines in colon. The color intensity showed the degree of correlation (blue represents a negative correlation, whereas red shows a positive correlation). *p < 0.05, **p < 0.01, and ***p < 0.001 (*n* = 8 or 12 per group).

### Multivariate ROC Analysis Demonstrates Robust Metabolic Discrimination across Experimental Groups

Multivariate ROC analysis was conducted to assess the discriminatory performance of metabolomic profiles among ND, DSS, HGAS, and AS groups using pairwise comparisons (Figure 8). For ND versus DSS, a three-metabolite model achieved an AUC of 0.949 (95% CI: 0.675–1), which increased to 0.993 (95% CI: 0.929–1) when five metabolites were included. Models incorporating ≥10 metabolites achieved an AUC of 1.0. For HGAS versus DSS, a three-metabolite model yielded an AUC of 0.997 (95% CI: 0.963–1), and inclusion of five or more metabolites resulted in an AUC of 1.0. For HGAS versus AS, the three-metabolite model achieved an AUC of 0.940 (95% CI: 0.778–1), improving to 0.989 (95% CI: 0.803–1) with five metabolites. Models including ≥10 metabolites again reached an AUC of 1.0. Feature selection frequency analysis identified tetracosanoic acid as a consistently selected metabolite across comparisons, indicating high stability and a contribution to group discrimination.

**Figure 8:**
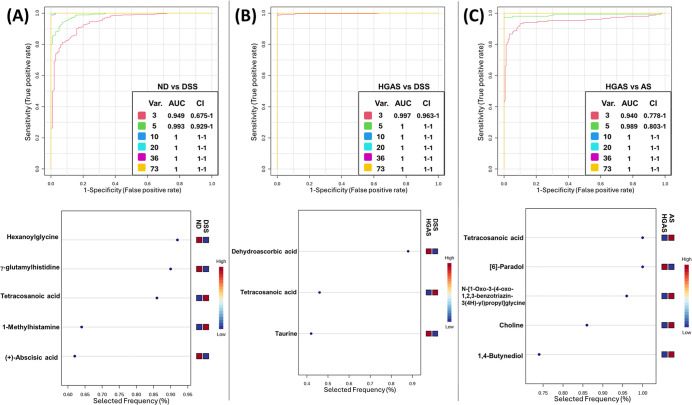
Multivariate ROC analysis and feature selection demonstrate robust metabolic discrimination among experimental groups. Multivariate ROC curves generated using Monte Carlo cross-validation (MCCV) with balanced subsampling for pairwise comparisons: (A) ND vs DSS, (B) HGAS vs DSS, and (C) HGAS vs AS. Classification models were constructed using increasing numbers of selected metabolites (3, 5, 10, 20, 36, and 73 variables). The area under the ROC curve (AUC) and corresponding 95% confidence intervals (CIs) are presented in each pane. Lower panels show feature importance and selection frequency (*n* = 8 or 12 per group).

## Discussion

The present study demonstrates that GAS markedly alleviates DSS-induced colitis through coordinated suppression of inflammatory signaling and broad modulation of systemic metabolic pathways. Compared with aspirin alone, GAS more effectively reduced colonic pro-inflammatory cytokines, suppressed COX-2 expression, improved disease activity parameters, and reversed a wide spectrum of DSS-associated metabolic disturbances. These findings suggest that GAS exerts protective effects that extend beyond classical cyclooxygenase inhibition and involve multipathway regulatory mechanisms.

At the tissue level, DSS administration induced robust inflammatory responses characterized by significant elevations in IL-6, TNF-α, and IL-1β, together with increased levels of COX-2 expression in colon tissue. GAS treatment markedly attenuated these pro-inflammatory mediators, particularly within the colon, where the magnitude of suppression exceeded that observed in plasma. This localized effect is consistent with the primary site of DSS-induced injury and suggests that GAS effectively modulates mucosal inflammatory responses. Although aspirin partially reduced certain inflammatory markers, its effects were less consistent and did not uniformly reach statistical significance. Given that aspirin exerts anti-inflammatory activity primarily through cyclooxygenase inhibition, these findings indicate that COX-dependent mechanisms of COX alone may be insufficient to fully counteract the complex inflammatory cascades triggered by DSS. In addition, aspirin is known to cause gastrointestinal mucosal irritation, which may further compromise barrier integrity in an acute colitis setting and attenuate its protective effects.

In contrast, the conjugation of aspirin with [6]-gingerol may mechanistically overcome these limitations. [6]-Gingerol has been reported to exert anti-inflammatory and antioxidant activities through multiple COX-independent pathways, including modulation of oxidative stress, inflammatory signaling, and epithelial barrier function. The GAS conjugate, therefore, may provide a dual benefit by retaining aspirin’s COX-mediated anti-inflammatory activity while simultaneously expanding the spectrum of protective mechanisms and mitigating gastrointestinal injury. This multitarget profile may explain why GAS consistently outperformed aspirin across multiple inflammatory and histological endpoints in the present study.

Beyond cytokine suppression, untargeted metabolomic profiling revealed extensive metabolic disruption following DSS treatment and substantial normalization after GAS administration. Multivariate analysis demonstrated clear separation between ND and DSS groups, reflecting systemic metabolic reprogramming induced by colitis. GAS shifted the metabolic profile toward that of normal controls, whereas aspirin-treated animals remained metabolically closer to the DSS group. Among the 217 metabolites significantly altered by DSS, 73 were significantly reversed by GAS, highlighting a broad metabolic response.

Classification of GAS-responsive metabolites into four response zones further revealed distinct regulatory patterns. While some metabolites were normalized by both GAS and aspirin, a substantial subset was selectively restored by GAS, suggesting regulatory effects beyond those attributable to aspirin alone. Lipid-derived metabolites constituted the largest category among the identified metabolites, underscoring the central role of lipid metabolism in DSS-induced metabolic dysregulation. Alterations were also observed in amino acid catabolism and carbohydrate-related pathways, indicating a coordinated perturbation of energy metabolism and oxidative processes during colitis.

Zone 1 consisted of metabolites that were elevated by DSS treatment and reversed by both GAS and aspirin. Representative metabolites in this zone included acetoacetic acid, d-xylose, d-galacturonic acid, glyceric acid, glyceraldehyde, and 3-hydroxyadipic acid 3,6-lactone. With the exception of 3-hydroxyadipic acid 3,6-lactone, these metabolites have been reported to be altered in clinical samples from patients with colorectal cancer (CRC) or inflammatory bowel disease (IBD).^11–19^ Notably, 3-hydroxyadipic 3,6-lactone is a recognized biomarker of fatty acid ω-oxidation,^20^ suggesting that GAS may also influence lipid oxidation pathways. The normalization of these metabolites by GAS indicates improvements in epithelial metabolic homeostasis and reductions in the level of oxidative metabolic stress.

Zone 2 included metabolites that were elevated by DSS treatment and selectively reduced by GAS. Key metabolites in this group included 5-oxoproline, choline, and tetracosanoic acid, all of which have been reported to be dysregulated in IBD or CRC.^11–15,21,22^ The restoration of these metabolites by GAS suggests the partial normalization of amino acid metabolism, membrane-associated choline metabolism, and lipid-related pathways that are commonly disrupted during intestinal inflammation.

Zone 3 included metabolites that were reduced by DSS treatment and restored by GAS and aspirin. Several representative metabolites in this zone—such as alanylglycine, hexanoylglycine, *N*-acetylleucine, and urocanic acid—have previously been reported to exhibit abnormal levels in patients with CRC.^13–15^ The reversal of these metabolites indicates that both treatments can modulate inflammation-associated disturbances in amino acid catabolism, although GAS generally produced a stronger normalization effect.

Zone 4 contained metabolites that were reduced by DSS treatment and selectively restored by GAS. The identified metabolites in this zone that have been previously associated with CRC or IBD^13,14,21,23–26^ included 3-hydroxyoctanoic acid, 4-hydroxynonenal, benzyl alcohol, and hexanoic acid. This differential regulation highlights distinct metabolic responses to GAS and aspirin treatments and further suggests that GAS may influence host metabolic homeostasis beyond its aspirin component. Consistent with this notion, metabolomic profiling revealed broader metabolic normalization in the GAS-treated group compared with aspirin alone, further supporting the concept that conjugation with [6]-gingerol confers multipathway modulation rather than a single-target effect.

Correlation analysis provided additional integrative insight. Sixteen of 17 representative metabolites exhibited significant associations with colonic cytokines, linking systemic metabolic changes with local inflammatory status. This two-layered observation—metabolite reversal by GAS combined with a correlation to inflammatory mediators—supports the biological relevance of these metabolic alterations. Nevertheless, these relationships remain correlative. Untargeted metabolomics is inherently hypothesis-generating, and the causal roles of individual metabolites require targeted quantification and functional validation.

Multivariate ROC analysis further demonstrated a strong discriminatory capacity of metabolomic signatures among experimental groups. Even models incorporating only three metabolites achieved high AUC values, suggesting that a limited metabolic panel may reliably distinguish between inflammatory and treatment states. Tetracosanoic acid emerged as a consistently selected discriminatory metabolite with a high stability across cross-validation analyses. While models incorporating larger metabolite panels achieved near-perfect classification performance, such results should be interpreted cautiously, given potential overfitting and sample size considerations. Independent validation in larger cohorts is necessary to confirm robustness.

The untargeted metabolomic profiling performed in this study identified 73 GAS-responsive metabolites that were significantly altered in the DSS-induced colitis model. While the normalization of these metabolites was associated with improved inflammatory outcomes, these findings should be interpreted as correlative rather than causal. Untargeted metabolomics is inherently hypothesis-generating and does not establish direct mechanistic links between individual metabolites and biological effects. Therefore, the identified metabolites should be considered potential biomarkers or candidate mediators that reflect GAS-associated modulation of inflammatory pathways rather than definitive drivers of the observed anti-inflammatory effects. Targeted quantification and functional validation will be necessary to determine their causal roles.

Mechanistically, the superior performance of GAS relative to that of aspirin may reflect the combined properties of its constituent moieties. Aspirin primarily modulates inflammatory responses through the inhibition of prostaglandin synthesis and downstream signaling pathways. In contrast, ginger-derived compounds such as [6]-gingerol are well documented to exhibit anti-inflammatory, antioxidant, and NF-κB–modulating activities^27^ and have been reported to attenuate gastrointestinal injury in preclinical studies.^28^ Conjugation of aspirin with [6]-gingerol may therefore expand the therapeutic spectrum by integrating the COX-dependent anti-inflammatory activity with broader metabolic and cytoprotective effects. The distinct metabolic signatures observed in GAS-treated animals are consistent with such multitarget modulation.

Several limitations of this study should be acknowledged. First, the experiments were conducted in an acute DSS-induced colitis model, which primarily reflects epithelial injury and innate inflammatory responses and does not fully recapitulate the chronic inflammation and tumorigenesis observed along the IBD–CRC continuum. Although the aspirin-equivalent doses used in this study were calculated according to FDA-recommended body surface area normalization and fall within the range of low-dose aspirin commonly prescribed in humans, extrapolation to clinical application and long-term chemoprevention should be made cautiously. The acute experimental design does not permit the assessment of sustained efficacy, tumor suppression, or long-term safety. Future studies using chronic DSS or AOM/DSS colitis-associated colorectal cancer models, together with equimolar or equipotent comparisons between aspirin, [6]-gingerol, and GAS, will be necessary to dissect the specific contribution of the conjugate and to evaluate long-term chemopreventive relevance.

Second, the DSS-induced colitis model inherently causes significant colonic epithelial injury and mucosal disruption. Although the successful establishment of colitis in this study was supported by colon shortening, fecal occult blood positivity, body weight loss, and marked increases in pro-inflammatory cytokines, histological evaluation of colon tissue was not performed. We acknowledge that histopathological analysis would provide important morphological confirmation of the disease severity and further strengthen the pathological assessment. Moreover, because DSS itself induces substantial epithelial damage, it is difficult to clearly distinguish whether the observed effects reflect an intrinsic gastroprotective property of GAS relative to aspirin or simply attenuate DSS-induced tissue injury. Therefore, definitive evaluation of the comparative gastrointestinal safety profile of GAS will require future studies using chronic administration models without DSS-induced injury, together with direct assessment of gastric and duodenal histology, bleeding parameters, and COX-1/COX-2 inhibition profiles.

Third, plasma-based metabolomics does not offer direct insight into luminal- or epithelial-specific mechanisms. Future studies incorporating colonic tissue metabolomics, fecal metabolomics, and transcriptomic analyses of colonic epithelium will be important to establish tissue-specific mechanisms and host–microbiota interactions underlying the observed protective effects.

Finally, the formal pharmacokinetic parameters were not determined in the present study; indirect evidence supports the *in vivo* bioavailability and metabolic conversion of GAS. Consistent with our previous report published in Scientific Reports,^3^ GAS remains stable under gastric acidic conditions and undergoes decomposition primarily in the intestinal environment or after absorption, leading to the simultaneous release of aspirin and [6]-gingerol. In the current study, plasma metabolomics analysis further substantiated this metabolic conversion. Specifically, known metabolites of aspirin and [6]-gingerol—including salicylic acid, salicyluric acid, 6-gingerol-4′-glucuronide, and 3R,5S-6-gingerdiol-4′-glucuronide—were detected in circulation (Supporting Information, Figure S2). Quantitative comparison of peak areas confirmed their presence in treated animals, supporting systemic exposure to the active components derived from GAS. These findings provide supportive evidence that GAS survives gastric conditions, is absorbed, and undergoes intestinal metabolism to release its bioactive constituents. Nevertheless, dedicated pharmacokinetic studies will be required to fully characterize parameters, such as absorption rate, bioavailability, and systemic exposure.

In conclusion, the combined biochemical and metabolomic findings indicate that GAS alleviates DSS-induced colitis through the coordinated suppression of inflammatory mediators and broad normalization of disrupted metabolic networks. This multilayered regulatory profile distinguishes GAS from aspirin alone and supports its potential as a multitarget anti-inflammatory strategy. Further mechanistic and long-term validation studies will be essential to clarify its translational relevance and chemopreventive potential.

## Supplementary Material



## Notes

Food and water intake during the animal experiment; plasma metabolites of aspirin- and [6]-gingerol following GAS administration (PDF)

## References

[1] Fisher M., Knappertz V. (2006). The dose of aspirin for the prevention of cardiovascular
and cerebrovascular events. Curr. Med. Res.
Opin..

[2] Feng Y., Tao L., Wang G., Li Z., Yang M., He W., Zhong X., Zhang Y., Yang J., Cheung S., McDonald F., Chen L. (2021). Aspirin inhibits prostaglandins to
prevents colon tumor formation via down-regulating Wnt production. Eur. J. Pharmacol..

[3] Zhu Y., Wang F., Zhao Y., Wang P., Sang S. (2017). Gastroprotective
[6]-gingerol aspirinate as a novel chemopreventive prodrug of aspirin
for colon cancer. Sci. Rep..

[4] Sheng Y., Wu T., Dai Y., Ji K., Zhong Y., Xue Y. (2020). The effect
of 6-gingerol on inflammatory response and Th17/Treg balance in DSS-induced
ulcerative colitis mice. Ann. Transl. Med..

[5] Kobayashi T., Siegmund B., Le Berre C., Wei S. C., Ferrante M., Shen B., Bernstein C. N., Danese S., Peyrin-Biroulet L., Hibi T. (2020). Ulcerative colitis. Nat. Rev. Dis Primers.

[6] Chassaing B., Aitken J. D., Malleshappa M., Vijay-Kumar M. (2014). Dextran sulfate
sodium (DSS)-induced colitis in mice. Curr.
Protoc. Immunol..

[7] Ohkawara T., Nishihira J., Takeda H., Hige S., Kato M., Sugiyama T., Iwanaga T., Nakamura H., Mizue Y., Asaka M. (2002). Amelioration of dextran sulfate sodium-induced colitis by anti-macrophage
migration inhibitory factor antibody in mice. Gastroenterology.

[8] Liu X., Sun Z., Wang H. (2021). Metformin alleviates experimental colitis in mice by
up-regulating TGF-β signaling. Biotechnol.
Histochem..

[9] Taghipour N., Molaei M., Mosaffa N., Rostami-Nejad M., Asadzadeh Aghdaei H., Anissian A., Azimzadeh P., Zali M. R. (2016). An experimental model of colitis induced by dextran
sulfate sodium from acute progresses to chronicity in C57BL/6: correlation
between conditions of mice and the environment. Gastroenterology and hepatology from bed to bench.

[10] Esquivel-Alvarado D., Zhang S., Hu C., Zhao Y., Sang S. (2022). Using metabolomics
to identify the exposure and functional biomarkers of ginger. J. Agric. Food Chem..

[11] Hong Y. S., Hong K. S., Park M. H., Ahn Y. T., Lee J. H., Huh C. S., Lee J., Kim I. K., Hwang G. S., Kim J. S. (2011). Metabonomic understanding of probiotic
effects in humans
with irritable bowel syndrome. J. Clin. Gastroenterol..

[12] Kolho K. L., Pessia A., Jaakkola T., de Vos W. M., Velagapudi V. (2017). Faecal and
serum metabolomics in paediatric inflammatory bowel disease. J. Crohns Colitis.

[13] Sinha R., Ahn J., Sampson J. N., Shi J., Yu G., Xiong X., Hayes R. B., Goedert J. J. (2016). Fecal microbiota, fecal metabolome,
and colorectal cancer interrelations. PLoS One.

[14] Goedert J. J., Sampson J. N., Moore S. C., Xiao Q., Xiong X., Hayes R. B., Ahn J., Shi J., Sinha R. (2014). Fecal metabolomics:
assay performance and association with colorectal cancer. Carcinogenesis.

[15] Brown D. G., Rao S., Weir T. L., O’Malia J., Bazan M., Brown R. J., Ryan E. P. (2016). Metabolomics and metabolic pathway networks from human
colorectal cancers, adjacent mucosa, and stool. Cancer & metabolism.

[16] Ehrenpreis E. D., Salvino M., Craig R. M. (2001). Improving
the serum D-xylose test
for the identification of patients with small intestinal malabsorption. J. Clin. Gastroenterol..

[17] Ponnusamy K., Choi J. N., Kim J., Lee S. Y., Lee C. H. (2011). Microbial
community and metabolomic comparison of irritable bowel syndrome faeces. J. Med. Microbiol..

[18] Azario I., Pievani A., Del Priore F., Antolini L., Santi L., Corsi A., Cardinale L., Sawamoto K., Kubaski F., Gentner B., Bernardo M. E., Valsecchi M. G., Riminucci M., Tomatsu S., Aiuti A., Biondi A., Serafini M. (2017). Neonatal umbilical cord blood transplantation halts
skeletal disease progression in the murine model of MPS-I. Sci. Rep..

[19] Jonas A. J., Lin S. N., Conley S. B., Schneider J. A., Williams J. C., Caprioli R. C. (1989). Urine glyceraldehyde
excretion is
elevated in the renal Fanconi syndrome. Kidney
Int..

[20] Tserng K. Y., Jin S. J., Hoppel C. L., Kerr D. S., Genuth S. M. (1989). Urinary
3-hydroxyadipic acid 3,6-lactone: structural identification and effect
of fasting in adults and children. Metabolism.

[21] Le
Gall G., Noor S. O., Ridgway K., Scovell L., Jamieson C., Johnson I. T., Colquhoun I. J., Kemsley E. K., Narbad A. (2011). Metabolomics
of fecal extracts detects altered metabolic activity of gut microbiota
in ulcerative colitis and irritable bowel syndrome. J. Proteome Res..

[22] Ni Y., Xie G., Jia W. (2014). Metabonomics of human colorectal cancer: new approaches
for early diagnosis and biomarker discovery. J. Proteome Res..

[23] Garner C. E., Smith S., de Lacy Costello B., White P., Spencer R., Probert C. S., Ratcliffem N. M. (2007). Volatile organic compounds from feces
and their potential for diagnosis of gastrointestinal disease. FASEB J..

[24] De
Preter V., Machiels K., Joossens M., Arijs I., Matthys C., Vermeire S., Rutgeerts P., Verbeke K. (2015). Faecal metabolite profiling identifies medium-chain
fatty acids as discriminating compounds in IBD. Gut.

[25] Ahmed I., Greenwood R., Costello B., Ratcliffe N., Probert C. S. (2016). Investigation of
faecal volatile organic metabolites
as novel diagnostic biomarkers in inflammatory bowel disease. Aliment. Pharmacol. Ther..

[26] Di
Cagno R., De Angelis M., De Pasquale I., Ndagijimana M., Vernocchi P., Ricciuti P., Gagliardi F., Laghi L., Crecchio C., Guerzoni M. E., Gobbetti M., Francavilla R. (2011). Duodenal and faecal microbiota of celiac children:
molecular, phenotype and metabolome characterization. BMC Microbiol..

[27] Ayustaningwarno F., Anjani G., Ayu A. M., Fogliano V. (2024). A critical review of
ginger’s (*Zingiber officinale*) antioxidant,
anti-inflammatory, and immunomodulatory activities. Front. Nutr..

[28] Nikkhah
Bodagh M., Maleki I., Hekmatdoost A. (2019). Ginger in
gastrointestinal disorders: A systematic review of clinical trials. Food science & nutrition.

