# Supplementary material for: Gastroprotective [6]-Gingerol Aspirinate as a Novel Aspirin-Derived Chemopreventive Agent Attenuating Colitis
Source: J Agric Food Chem. 2026 Apr 20;74(16):12918–28. doi: 10.1021/acs.jafc.5c17778 (PMC13133900; doi:10.1021/acs.jafc.5c17778)
Supplement: Supplementary file 1 [file jf5c17778_si_001.pdf]

Supporting Information

Gastroprotective [6]-gingerol aspirinate as a novel aspirin-derived chemopreventive agent attenuating colitis

Pei-Sheng Lee[1], Shuwei Zhang[1], Yingdong Zhu[1], Vadin Ha[1], and Shengmin Sang[1,2,*]

[1]Laboratory for Functional Foods and Human Health, Center for Excellence in Post-Harvest Technologies, North Carolina Agricultural and Technical State University, Kannapolis, North Carolina 28081, United States

[2]Center for Gastrointestinal Biology and Disease, University of North Carolina at Chapel Hill, Chapel Hill, North Carolina 27599, United States

* All correspondence should be addressed to:

Dr. Shengmin Sang

E-mail: ssang@ncat.edu

Tel: 704-250-5710

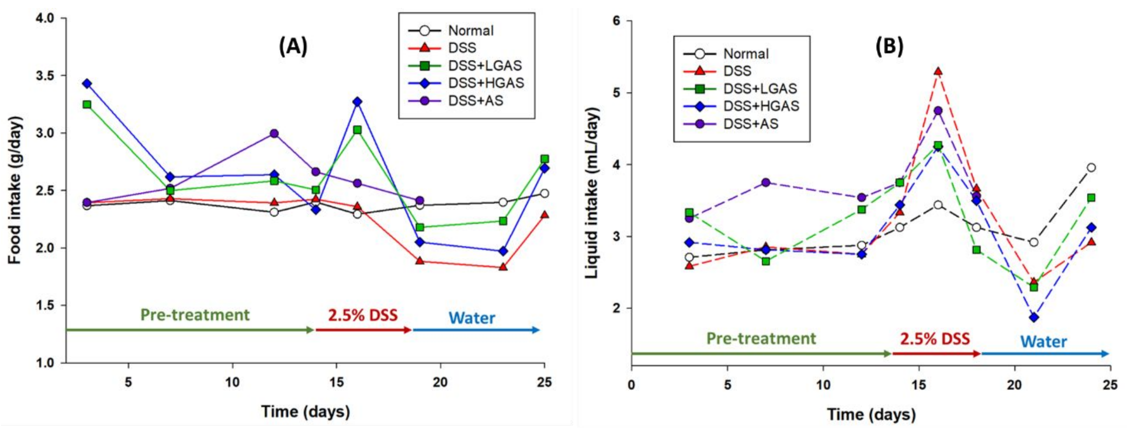

Figure S1. Food and liquid intake during the experiment period in DSS-induced colitis in mice. (A) Food intake; (B) Liquid intake.

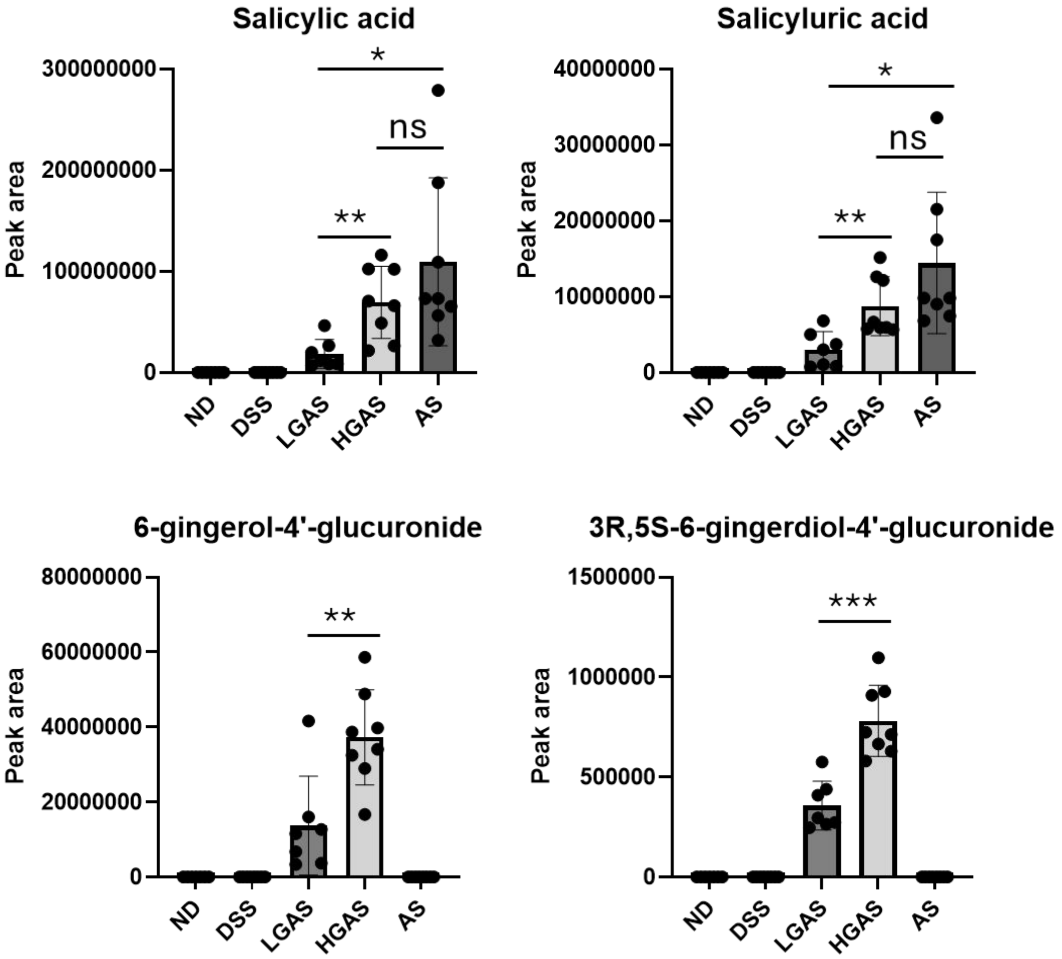

Figure S2. Plasma metabolites derived from aspirin and [6]-gingerol following GAS administration. Plasma levels of four representative downstream metabolites of GAS were analyzed by metabolomic profiling. Relative abundance is presented as peak area intensity. Salicylic acid and salicyluric acid represent major metabolites of aspirin, whereas 6-gingerol-4′-glucuronide and 3R,5S-6-gingerdiol-4′-glucuronide are characteristic metabolites of [6]-gingerol. Data are presented as mean ± SE (n=8 or 12 per group).
